# IVAN: Intelligent Van for the Distribution of Pharmaceutical Drugs

**DOI:** 10.3390/s120506587

**Published:** 2012-05-18

**Authors:** Asier Moreno, Ignacio Angulo, Asier Perallos, Hugo Landaluce, Ignacio Julio García Zuazola, Leire Azpilicueta, José Javier Astrain, Francisco Falcone, Jesús Villadangos

**Affiliations:** 1 Deusto Institute of Technology (DeustoTech), University of Deusto, Bilbao 48007, Spain; E-Mails: asier.moreno@deusto.es (A.M.); ignacio.angulo@deusto.es (I.A.); hlandaluce@deusto.es (H.L.); i.j.garcia@deusto.es (I.J.G.Z.); 2 Electrical and Electronic Engineering Department, Universidad Pública de Navarra, Pamplona 31006, Spain; E-Mails: leyre.azpilicueta@unavarra.es (L.A.); francisco.falcone@unavarra.es (F.F.); 3 Mathematics and Computer Engineering Department, Universidad Pública de Navarra, Pamplona 31006, Spain; E-Mails: josej.astrain@unavarra.es (J.J.A.); jesusv@unavarra.es (J.V.)

**Keywords:** intelligent van, pharmaceutical drugs traceability, incidences reporting, non-intrusive, RFID, wireless technologies, deterministic 3D ray launching

## Abstract

This paper describes a telematic system based on an intelligent van which is capable of tracing pharmaceutical drugs over delivery routes from a warehouse to pharmacies, without altering carriers' daily conventional tasks. The intelligent van understands its environment, taking into account its location, the assets and the predefined delivery route; with the capability of reporting incidences to carriers in case of failure according to the established distribution plan. It is a non-intrusive solution which represents a successful experience of using smart environments and an optimized Radio Frequency Identification (RFID) embedded system in a viable way to resolve a real industrial need in the pharmaceutical industry. The combination of deterministic modeling of the indoor vehicle, the implementation of an *ad-hoc* radiating element and an agile software platform within an overall system architecture leads to a competitive, flexible and scalable solution.

## 1. Introduction

Intelligent transportation systems (ITSs) improve transportation safety and mobility while enhancing productivity through the integration of advanced communications technologies into the transportation infrastructure and in vehicles [1]. In-vehicle intelligent transportation systems are emerging as a major force in mobile communications, where automotive, communications, computer, and software are elements which cooperate among themselves in order to minimize the delivery time to market of the product while maximizing quality in the transportation of sensitive goods.

In particular, the pharmaceutical drug supply chain, from an economic and health perspective, requires controlling all stages of distribution: from the production phases of the drugs in the laboratory until they reach the pharmacies. This requirement is reflected by the Ministry of Health and Consumption of Spain through the new Royal Decree on drugs traceability, redacted in accordance to Directive 2003/94/EC of the Commission of the European Communities [2].

Adapting to the changes required by regulation imposes severe changes in the business model of different actors involved in the pharmaceutical sector. Moreover, associated costs are hardly feasible in a highly competitive industry where profit margins are often set by public administrations.

The features of the existing application scenario in drug delivery from the warehouse to the pharmacy pose particular challenges. The huge competition between pharmaceutical distributors forces them to preserve a high quality of service in terms of delivery time and reliability. Inside a market where all competitors are offering the same products at similar prices, service quality is a key factor because an incomplete or incorrect shipment can result in the loss of a client. These requirements fall on the carrier which is required to complete the route in a minimum time without making any mistakes during delivery. For these reasons it is compulsory to deploy a system that meets the new regulations that does not complicate current tasks of carriers. The installation of a system that requires the carrier to use hardware devices such as handheld RFID readers or Datamatrix adds new tasks in an inherently stressful job. Generally, each distribution route is travelled at least four times a day and a significant delay or wrong delivery often results in the loss of a pharmacy as a client. Therefore, the job of the carriers should be thoroughly reviewed to avoid mistakes and if they commit to defined responsibilities, causing significant disagreements between transport staff and store personnel.

This paper presents a novel system approach designed to adapt the distribution of drugs to the new regulatory environment that does not alter the behavior of workers involved in it. The onboard system in each delivery van will collect all the information required for traceability without any human interaction, with the aid of an embedded RFID system, which has been optimized in order to take into account the complex wireless nature of an enclosed vehicle with a traceable load. An in-house three dimensional deterministic ray launching code has been employed in order to model the indoor vehicle environment, leading to optimal layout of RFID elements, as well as to the later design of a specific antenna element. Furthermore, this system is responsible for validating all actions which vary the cargo during development of a route by interfering with the activity of the carrier only if it detects a deviance with respect to planning. The overall result is an implementation of a Smart Environment, which combines adaption of the wireless scenario and a flexible system architecture to support real time monitoring and interaction. Thus, it represents a successful experience of using Ambient Intelligence (AmI) environments in a viable way to resolve a real logistic need [3]. One of the key elements in the performance of the overall system is the holistic approach, in which precise modeling of physical layer aspects is combined with a novel and flexible software architecture.

This paper is divided into three main parts. Initially the functional characteristics of the system presented are indicated. The Intelligent Van system is described, in which an embedded RFID system is proposed, which increases the reading performance and therefore the traceability capabilities. The data related with the delivery route is controlled and compared by means of the System Architecture, which consists of the necessary middleware as well as with a user friendly application which is resident in a Smartphone device. Finally through the conclusions we will show the results of implementing the system in a real warehouse for distribution of pharmaceuticals located in the north of Spain.

## 2. Intelligent Van

The intelligent container concept has been envisaged in logistics and distribution processes. However the specific needs of the scenario corresponding to the distribution chain of medicines requires the development of a customized solution. Furthermore, the high cost of RFID tags imposes restrictions in the implementation of such systems in real applications, especially in scenarios with heterogeneous loads [4]. The use of tags in the 13.56 MHz HF frequency range with reduced reading distance, limits the identification of the packets to the moment in which they are loaded or unloaded. Therefore, the proposed system uses long-distance UHF tags in order to monitor the vehicle cargo at any time. This extension in the reading range is given by the fact that interaction of RFID information is not given by mutual inductance mechanisms, but instead is determined by the modification of the radiofrequency wave, in terms of the radar cross section of the given tag [5].

Two of the main characteristics of this system are the possibility of controlling medical distribution over the complete delivery route and to simplify carriers' workflow. In order to achieve these goals, an intelligent system has been established introducing Radio Frequency Identification technology (RFID) in pharmaceutical drug distribution. This technology is optimal in order to determine the traceability of all drugs delivered and enables the drivers to load and unload the cargo due to the fact that if any error is committed, the system will alert indicating the failure in an autonomous way.

Focusing on the general process, initially automated medical dispensing robots within the main distributor depot have to coordinate orders for each pharmacy. This system organizes all requested medicines in containers and then all of them are sent to the dock ready to be loaded onto distribution vans. There is a passive transponder attached to each container so its Electronic Product Code (EPC code) can be related to what drugs are loaded on each container and the pharmacy destination for each one. Within the regulatory framework of Spanish law, traceability can be established with the use of two technologies, Low Frequency RFID and DataMatrix for the identification of individual packages of medicines. A second phase of this project that will begin in the second quarter of 2012, will implement a robot that can dump the contents of a container and validate, based on identification technologies approved, the packaging of medicines contained within. Thus the system will meet the requirements of traceability required by Spanish law.

**Figure 1.**
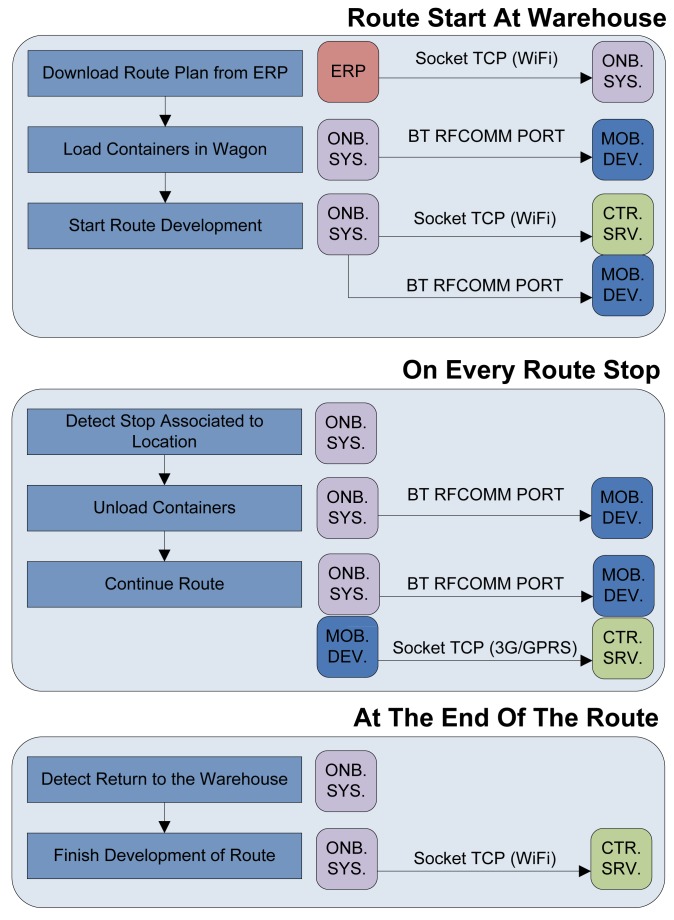
Process flow diagram of the system.

Figure 1 shows the process flow indicating the communication established between the three main parts of the system: onboard system (ONB SYS), mobile device (MOB DEV) and central server (CTR SRV). When the delivery van arrives at the warehouse, the onboard system installed on each van is connected to the network of the warehouse via WiFi and is allowed to download from the corporative Enterprise Resource Planning (ERP), all the necessary information about the next route the vehicle must perform. This info includes the number of containers that must be loaded into the cargo area of the Intelligent Van and the Electronic Product Code (EPC) of each transponder attached to containers that have to be distributed along the route.

At this point, carriers start to load vans with their corresponding containers. An RFID reader module is located inside the wagon and each container has attached a passive tag so that it can detect each container that enters or leaves the wagon. It is very important to emphasize that this is a non-intrusive system. The carrier does not have to be concerned about registering loaded or unloaded containers due to the fact that the RFID environment of the proposed system will do it automatically. The RFID module detects transponders EPCs and transmits them to the onboard system. This device stores all the information needed for a correct delivery and monitors what the carrier is doing at any time.

If the carrier makes a mistake loading or unloading a wrong container a red light will switch on in the cargo area of the Intelligent Van and the carrier will know that there is something wrong. Since all van drivers carry a Smartphone with them, a specific application has been designed, so the Smartphone is considered an element of the proposed system. That mobile is connected by Bluetooth to the van's onboard system, having precise knowledge of the list of containers to load into their vans and which containers are being loaded or unloaded. Therefore when the red light switches on, the carrier will get an error message generated by the proposed system on his Smartphone. The driver will try to solve it and in the case that it is not possible to be resolved, the Smartphone will send an incident report to the warehouse manager and that incident will also be registered on the onboard system. The mobile application is resident on the Smartphone and is only accessed by the carrier when the red light indicates an error, so the application shows a description of occurred mistake. In the event that the development of the transport process is executed as planned the carrier does not need to open the application and adds no new tasks to his usual workflow.

Once the cargo is loaded, the van driver starts the delivery. Each driver has a route of pharmacies where some containers must be unloaded at each time. With the aid of a Global Position System (GPS or future Galileo System) transceiver incorporated within the onboard system, the van knows its rough location at any given time. Therefore, the intelligence of the system calculates which containers to unload in the next pharmacy as well as in the remaining stops within the route. When the van stops and the cargo door is opened, the system detects the nearest pharmacy included in the route accessing which containers should be unloaded at this point. When the carrier unloads all containers associated with that stop and no other, the system activates the green light, indicating that unload procedure is correct. If after downloading all containers, the red light remains on, the carrier must check the mobile application to detect the cause of the error. If there is any divergence that cannot be solved, the carrier can continue to the next stop and the system immediately reports an incident to the warehouse manager.

As it is a non-intrusive system, the carrier performs the workflow in the usual fashion, not realizing that behind there is a system controlling every event and being notified only in case of an error. The system in addition includes atmospheric sensors inside the cargo area of the Intelligent Van for temperature, pressure and humidity control of the transported goods, with the capability of introducing new sensors if necessary.

The onboard system stores in a Secure Digital (SD) card, at regular configurable intervals, the location of the van, EPCs of containers loaded and the values of the atmospheric sensors. When the route ends and the van returns to the pharmacy warehouse, the system connects to the network of the warehouse via WIFI and sends an XML file through File Transfer Protocol (FTP) containing all the information stored during the development of the route. This allows the warehouse manager to subsequently review the development of a route through the control panel and search for causes of any errors and delays.

Comparing the key points of our research with other works related to distribution and traceability [6,7] it must be pointed out that the proposed solution in this paper provides innovations that are not accomplished in other proposed traceability systems, such as the ability to perform the cargo tracking and the monitoring of the transport tasks without the aid of the carrier and in a completely non-intrusive way. Most of the existing solutions are only aimed at the traceability of the cargo either manually or automatically [8], but do not have enough intelligence to propose autonomously the tasks to be performed along the way from receipt of order. This potentially leads to a lack of precision in the information provided by these traditional tracking systems as well as in the generated incidents.

## 3. Cargo Identification System

According to the World Health Organization (WHO) drugs must be stored and transported under specific environmental conditions as well as security constraints in the drug delivery process. One of these conditions is the need to use standardized and reusable containers to transport those drugs from warehouses to each pharmacy office. This fact constitutes a suitable scenario for RFID technology and this project in particular, enabling the possibility of controlling which drugs go to each pharmacy if they are contained within a registered recipient. The system is capable of determining the place where containers have been left, supported by a GPS or Location Based receiver. The attachment of a passive tag to each container, not only allows the system to know when containers enter or leave the cargo area of the Intelligent Van, but also makes the investment in tags affordable in a very short time, reducing return of investment cycles, avoiding one of the biggest drawbacks in the adoption of this technology. Carrying drugs into containers that have a transponder attached also allows tracking and traceability of them.

The intelligent system designed is based on several technologies, being RFID the most relevant in the physical layer interaction. This technology uses two basic elements, a tag or transponder and a reader or interrogator. As already mentioned, passive transponders have been attached to containers exploiting its reusability, and an RFID reader has been placed inside each van. To maximize the reader's gain, several tests have been made with different antennas, custom designed and commercial antennas, and with different antenna locations, considering the complexity of the wireless environment of an enclosed vehicle.

### 3.1. RFID Tag

**Figure 2.**
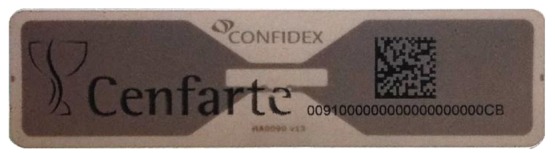
Confidex's Carrier Tough passive tag.

A Confidex's Carrier Tough (Figure 2) tag has been chosen to be attached to a container. It is a passive tag (*i.e.*, no energy sources) covered in hard plastic and resistant to mechanical stress, friction and shock. It also has a paper with a 2-D Data Matrix that is useful for other applications in other parts of this supply management.

The tag works using EPC Gen2 Class1 protocol, its frequency range is from 860–960 MHz and its reading range is from 4 to 6 m, enough for a wagon van. The price of tags is decreasing year by year, but it still remains a problem for many companies to adopt this technology. The reusability of these transponders thanks to the fact that they are fixed on the wall of the containers and not on each drug box makes RFID an adequate technology for the project because of its easy investment recovery.

### 3.2. RFID Reader

In order to enable interaction between the system and the containers, an RFID reader is located into each delivery van, which is permanently in contact with the embedded device next to it. The embedded device acts as a control and storage device and tells the reader to read or write tags and send to it received EPCs of the transponders inside the wagon when the system needs it. This reader module has RS-232 communication on one side with the embedded device, and wireless communication with the passive transponders on the other side.

A Thing Magic's Mercury5e-EU RFID Reader [9] has been employed, operating at UHF frequency range to improve interrogation distance and bears EPC Gen2 protocol, more robust against noise and reading interferences. It has 30 dB read gain at the range of 865.6–867.6 MHz according to the European Union regulatory support ETSI (EU) EN 302208. With an antenna of at least 6dBi, it can read tags in 9 m within its nominal sensitivity of −65 dBm.

The operational procedure of this system starts with the embedded device. It interrogates for the information of tags within the cargo area of the van by sending a request through serial port. The RFID reader starts generating a continuous wave to power up those tags and they backscatter their EPC codes (96 bits) modulating the continuous wave generated by the reader [10]. To detect all those backscatter signals generated by transponders [11], a high gain antenna is connected to the reader and is strategically located into the van, in order to minimize the link balance established between the tags and the reader. In this manner the reader improves its reading range limited by the low power response of transponders, increasing wireless coverage inside the vehicle. Once it has completed a read cycle, the reader sends back through serial port all EPCs stored in its internal buffer to the device.

For tests, some useful parameters have been used to improve detection of the tags. When the reader communicates with a tag it stores the received signal strength indicator (RSSI) of the tag read, as well as the time the tag was read, relative to the time the command to read was issued (Timestamp). These parameters have been employed in order to locate the antenna to get the best tag detection results.

### 3.3. RFID Antenna

The coverage range of the indoor RFID system is given by the bi-directional link balance of the wireless channel, in which parameters such as antenna gain, cable and feeder losses and a combination of losses due to interaction of electromagnetic waves with the indoor vehicle environment determine the final result [5]. Initially, from the hardware point of view, to enhance coverage over the wagon an antenna for the reader is needed. As it has been stated, with a 6 dBi antenna, the cargo section of a standard van can be covered. Validation tests and simulations have been made with different commercially available antennas of at least 7 dBi of gain, as well as with an antenna that has been designed specifically for this application within the research group.

Simulations have been made using the van as scenario to analyze commercial antennas' behavior. The wagon has been modeled as a metallic cube full of polypropylene containers and the antenna has been placed at different locations in the ceiling of the van. Simulations are based on the deterministic method of a 3D beam source, with an in-house ray launching code [12] to analyze the complex scenario of a wagon full of containers. It is interesting to stress that the topology and morphology of the indoor cargo section of the vehicle have a significant impact in the response of the system. This is given by the fact that strong multipath is present, as well as diffuse scattering due to the different levels of detail of the surfaces of the vehicle walls, as well as the transporting bins. An additional consideration is the fact that the transporting bins are mainly made of dielectric material (polypropylene), leading to new diffractive components as well as absorption losses. Therefore, an adequate simulation technique has to be applied in order to obtain propagation losses and consequently, link balance results.

**Figure 3.**
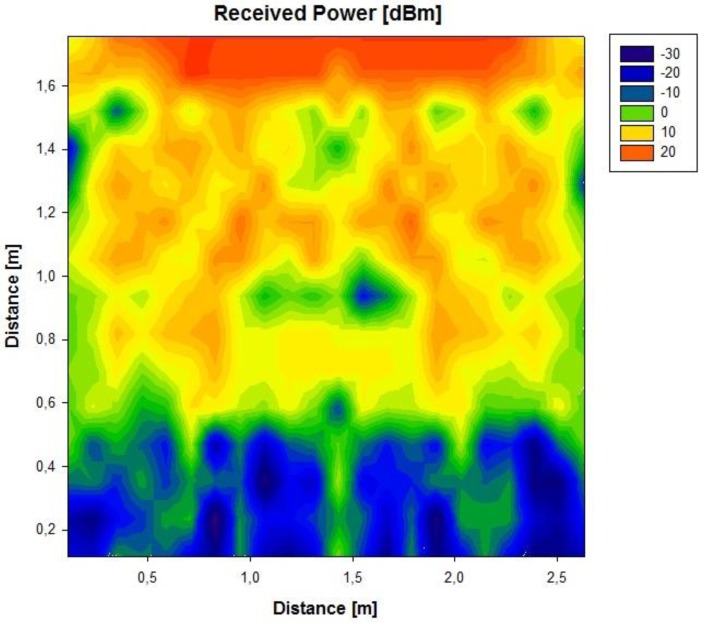
Estimation of received power [dBm] on the second floor of containers, obtained by full 3D ray launching algorithm.

**Figure 4.**
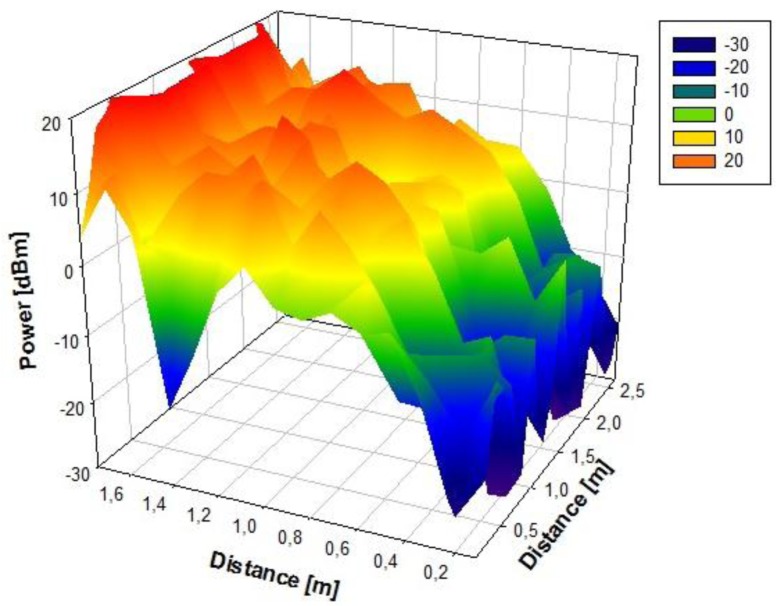
Volumetric view of simulated values of received power [dBm] on the second floor of containers seen from the side.

Initially, a linearly polarized PATCH-0026 and a circularly polarized PATCH-0025 have been used, which exhibit very similar parameters except for their polarizations. In this way, both antennas have a similar reading range behavior because the wagon is a closed metallic environment so there is multipath propagation. The RFID reader will receive a direct component, if there is direct visibility, or there will be a great number of echoes with different amplitudes, phases and random arriving times. The circularly polarized antenna has been chosen because it has the best coverage over the wagon. Figures 3 and 4 show the power distribution inside the wagon using the latter antenna. The results have been obtained with the aid of an in-house 3D ray launching code implemented within our research group, which takes into account reflection, refraction and diffraction phenomena, as well as the material properties (given by dielectric constant values as well as conductivity values at the operational frequency of the system). This simulation technique is optimal in the terms of precision *versus* computational time, due to the deterministic nature of the simulation and the geometrical optics approximation. Moreover, the implemented code has been optimized in order to take into account the complex nature of the vehicle. Figures 3 and 4 depict an estimated power received by likely tagged containers that are located at the second row of the packing distribution and that in an isotropic view, respectively. It has been placed at the back of the van in the middle of the ceiling offering the optimal results of the simulations performed. Due to its polarization, tags can be read in any orientation. In an environment like the one given by the indoor region of the cargo area of the van, the transmission power decreases with distance with great variability, due to the strong influence of multipath components.

### 3.4. Antenna Design

To maximise space inside vehicles and contribute to a less bulky UHF-RFID interrogator unit, an novel antenna was implemented to satisfy the demands of the proposed system. Spraying adhereable copper particles [13,14] to form conductors leading simple structures and manufacturing of antennas suited for use in radio frequency identification networks using the unlicensed RFID subband b2 (8.656–8.676 GHz) of the ETSI standard [15] are presented. The on-going prototype presents advantages such as consistency against likely antenna instability while on-the-move [16], is inherently hidden to account against fraudulent hacking and exhibits a small footprint, leading to an overall compact device.

On-going studies suggest that the antenna can be also used as a tag at a −5 dB return loss (RL), providing available transponder chips with characteristic impedances in the range of ∼12.50 + j17.30; this would bring applications for tagging complete metal shielded objects in a wide range of situations, such as boxes, bottles and cans, typically used for the transport of liquid based medicines with advanced care.

**Figure 5.**
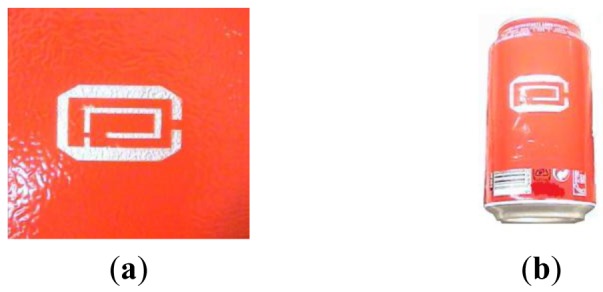
(**a**) The antenna printed on a car body and (**b**) printed on a can body.

**Figure 6.**
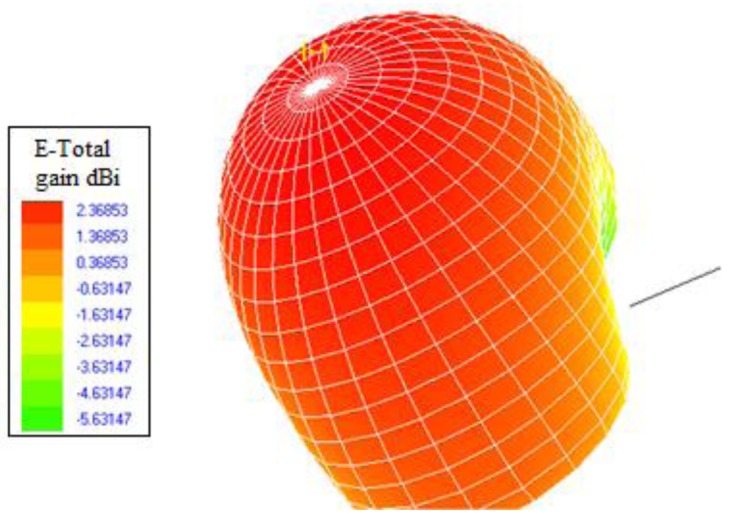
The radiation pattern of the proposed embedded RFID antenna, E-Total, showing simulated gains, within the 2 dBi range.

**Figure 7.**
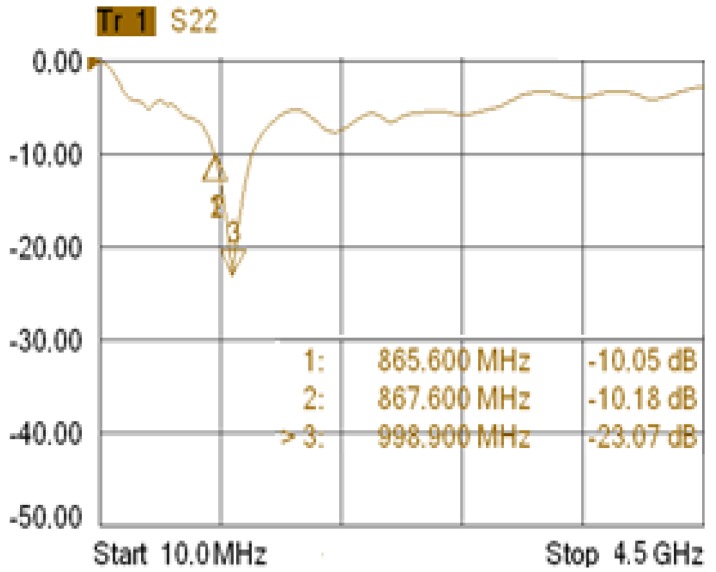
Measured return loss of the proposed embedded RFID antenna.

The geometry of the manufactured antenna for both the interrogator and the tag is presented in Figure 5a,b, respectively. For the approach the total substrate thickness of the paint was 2 mm. The antennas were made using conductive copper painting and directly printing the elements onto the surfaces (*i.e.*, vehicle body and can body). The achievable gains and RL of the antenna are given in Figures 6 and 7 respectively, showing sufficient gain and bandwidth for the application.

**Figure 8.**
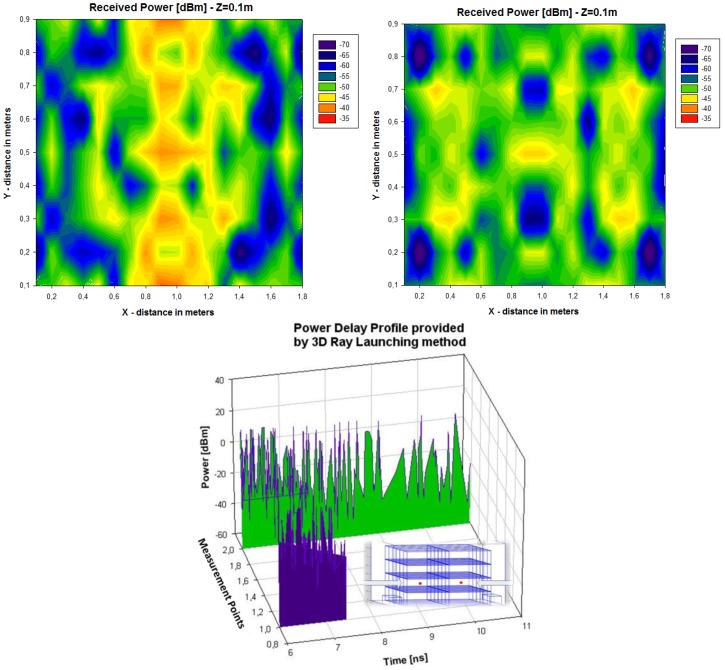
Simulation result of the resulting power distribution within the enclosed vehicle environment, obtained by in-house 3D ray launching code. The left hand figure is the result for the new embedded antenna, whereas the right hand figure corresponds to the conventional one. The bottom figure depicts estimated values of Power Delay Profiles for two different positions within the indoor cargo area of the Intelligent Van.

Once the antenna parameters have been obtained, an analysis of the resulting link balance has been carried to determine acceptable antenna gains for the in-vehicle and 2 dBi seems to be adequate for the application; relatively low gain antennas imply smaller sizes and is seen attractive for physically optimising the antenna size. It was found that antennas with gains of 2 dBi and located in the middle of the ceiling of a vehicle are adequate to deliver a sufficient radio propagation field inside the vehicle with full power transmission at the transceiver [5]; any superior power beyond the total transmit power of +30 dBm would not be acceptable by the ETSI standard for in-door applications [15]. The antenna ensures good power distribution to likely RFID tag locations within the car while minimizing field exposure to potential occupants [17] (those assisting in the inventory distribution of goods). The resulting enhancement by using the novel antenna design is shown (Figure 8), where deterministic ray launching results, by means of in-house simulation code, have been obtained as compared with a commercial antenna for the cargo section of the vehicle. The power distribution reveals higher received power levels, which leads to improved interrogation distance from the in-vehicle RFID reader. It is worth noting that full complexity of the environment has been taken into account (*i.e.*, structure of the van enclosure as well as the existence of the transporting bins). To further highlight the complexity of the indoor cargo area environment and the strong topological dependence, Power Delay Profile (PDP) values have been estimated with the aid of the 3D Ray Launching algorithm for two different locations (bottom image, Figure 8). The PDP values, which is a metric to determine the influence of multipath propagation, is clearly different in both positions, which implies the need of precise radioplanning techniques in order to fully optimize the behavior of the deployed RFID system.

## 4. System Architecture

**Figure 9.**
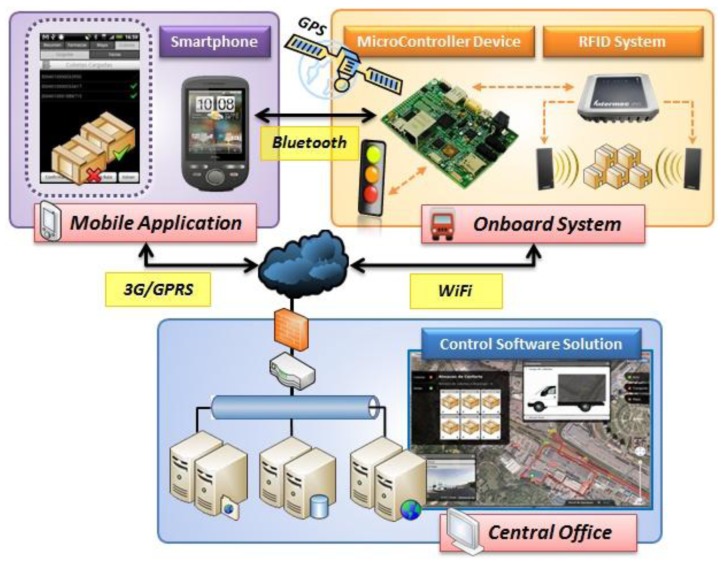
Description of the system architecture and possible interactions.

In order to fully exploit the benefits of the embedded RFID system, real time interaction with inventory as well as management is given by means of a supporting communication system. The core of the ubiquitous computing environment is given by the onboard system installed in the van. A specific communication middleware has been developed to connect this onboard system with the rest of modules that compose the system architecture (Figure 9). Taking advantage of the information exchanged with the rest of the systems and their additional processing capability, data gathered inside the vehicle can be enhanced. This becomes the van intelligent enough to report incidences in a completely autonomously way (without the aid of the carrier).

This system can be decomposed into three different parts: the onboard system itself, responsible for bringing together the information obtained by the hardware components of the van; the mobile application and the control software solution. In this section we are going to describe each module of the system from a functional and technical point of view.

### 4.1. Onboard System

To deploy this solution it is necessary to integrate an onboard system in each vehicle in the distribution fleet. This system is responsible for identifying the load, validate transport tasks and communicate every incident that is detected. Therefore, the onboard system is the core of the intelligent cargo solution. When a vehicle enters the loading dock to start a new route, the onboard system connects to the warehouse central server via WiFi system in order to download an XML file that includes all necessary information about the route the vehicle will perform. This file updates the pharmacies in the route, their location and the EPC codes of the containers to be distributed.

Whenever the carrier opens the doors of cargo section of the vehicle, the system activates the RFID reader that performs continuous readings for validating the loaded containers. Through two lights, green and red, the system indicates whether the transport actions are executed properly. The green light indicates that the containers loaded on the vehicle conform to the current position, while the red light reports a deviation from the planned actions. Due to the uploaded data about the routes to follow, the sensors embedded within the vehicle and the integrated geopositioning capacities, the device also provides the van driver the necessary knowledge to optimize routes and therefore, the quality of the service. This procedure ensures, in a non-intrusive way, the proper execution of the routes assigned to the van driver. This is done taking advantage of the intelligence provided by the various hardware components involved (antennas, sensors, tags, *etc.*) intercommunicated via wireless technologies (WIFI, Bluetooth, RFID) and its associated information system. All these elements conform the system architecture of the proposed ubiquitous computing solution.

The main device used to implement all these features is an ISEE IGEPv2 MPU platform based on a DM3730, a System on a Chip (Soc) that integrates 1GHz ARM Cortex-A8 Core. This is a small size card (93 × 65 × 15 mm) that has all the communications modules and resources demanded by the project. Embedded platform runs under a Linaro distribution with a Linux kernel optimized for this specific board. It includes WIFI IEEE802.11b/g communication capability used for updating information at the warehouse and a Class 2 Bluetooth 2.0 module, capable of communicating with the mobile application of the driver. The platform provides several Global Purpose Input/Ouput pins (GPIO) that are used to activate red and green lights and detect when the vehicle wagon doors are opened. Furthermore, this board has two serial ports (UART type) which are used for communication with the RFID reader and an external GPS receiver (Fastrax i310). Finally, the embedded platform has an SD card that stores the historical data acquired during the development of the route that are subsequently transferred to the central server. The onboard system is powered through an independent battery which is charged from the supply system of the vehicle when the engine is running.

**Figure 10.**
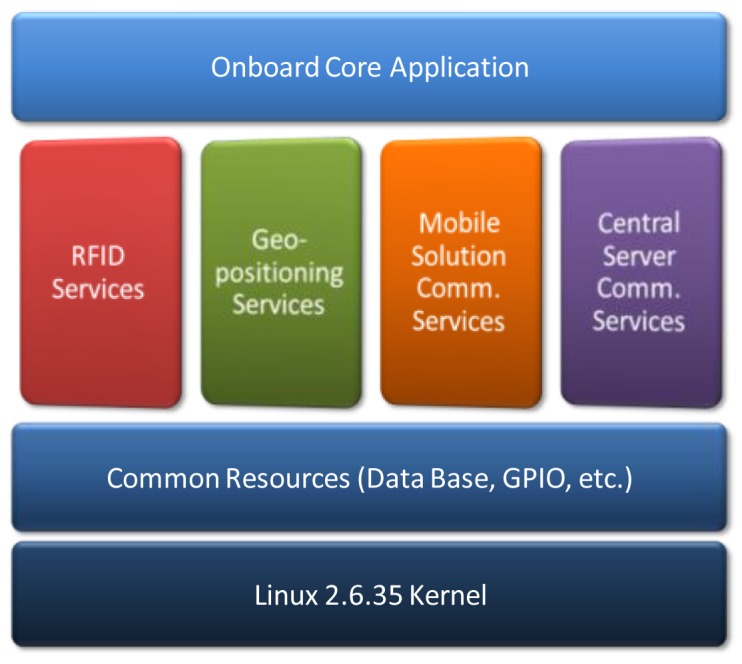
Middleware main modules.

To facilitate future portability of the developed solution to other scenarios within the freight transport, a middleware has been developed through the use of static libraries that allow the programmer to access easily to key project resources. Thus the firmware programmer can be abstracted from the specifics in communication protocols with distributed modules and focus on application functionality. Figure 10 shows how the middleware is divided into four main libraries which provide the following services:

- *RFID services*: Provides functions to read locatable tags, verify access to a particular label and configure the power of the reader drive.
- *Geopositioning services*: Includes resources for locating the vehicle, calculate distance between different points and find the nearest stop on route.
- *Communication services with mobile device*: Includes authentication resources and functions for receiving and sending main communication frames.
- *Communication services with central server*: Provides functions for reading and unpacking the file route planning, dispatching urgent incidents and packaging and sending of developed route file.

In deployment phase of the project the use of middleware will facilitate the replacement of peripherals used, allowing replace the RFID reader with a more economic one or GPS receiver with a more precise Global Positioning System without changing the core application. Similarly, integration with other ERP systems typical of other scenarios will require only the development of the specific module of the Middleware.

### 4.2. Mobile Application

The following extension of the system is the mobile application, which is installed on a Smartphone device. The van driver uses it to interact with the onboard system using a user-friendly graphic interface. The integration of a mobile device in the environment of the proposed solution can complement the system, serving the driver as an entry point to the knowledge offered by the system in a more direct and comprehensive manner [18], always maintaining the desired level of non-intrusiveness. This communication between the mobile device and the onboard system is established using Bluetooth technology at the start of each route.

Android OS has been used for the development of the mobile application. This decision was motivated by the opportunity offered by the pharmaceutical transport company CENFARTE (Centro Farmaceútico del Norte, S.A.) with whom an active collaboration has been established to enable the implementation of a real pilot of the solution. The company has the means to have an Android terminal for each route or carrier. As a result, the driver will carry a Smartphone to stay connected to the server application in order to know all specific needs on every transport service. The wide range of terminals and the variety of operating systems has forced us to develop a multiplatform application that works on the majority of newest existing smartphones, including Android OS, Apple iOS and BlackBerry OS. To achieve this goal, the development has been focused towards a Service Oriented Architecture (SOA), in which most of the functionality is distributed in the server, freeing the mobile devices of processing load, that will access the logic through SOAP messages to a web service developed for the control software solution.

**Figure 11.**
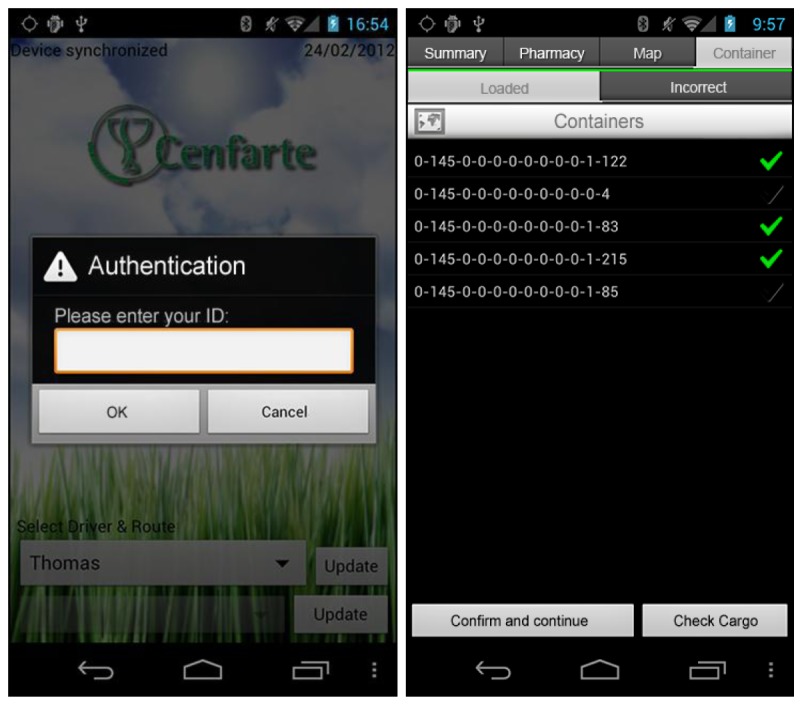
Developed mobile application interface.

Once transportation is available to perform a delivery service, the mobile application displays the routes that are currently available. It does so through a WiFi connection to the server, accessing the web service responsible for obtaining the daily routes not yet started. In case of failure of the WiFi connection, the system offers continued support in communications through GPRS/HSPA connectivity, available in the mobile device. Once the route is established, the mobile device obtains data about the full path: distance, estimated duration, number of stops, addresses, *etc.* Similarly, for each of the established halts relating to the pharmacies on a route, specific EPC codes of containers to download are obtained. This information is accessible via another web service that fulfills the function of data-oriented middleware enabling the capture of real-time information from the ERP system implemented in the company. The developed system obtains the information in a transparent and non-intrusive manner, not being necessary expensive modifications in the legacy order management system. All this information is displayed to the carrier through a user interface designed with the purpose to enable maximum usability and minimize intrusiveness (Figure 11).

The application core functionality is the management of incidents, giving as an added value support for transport activities and navigation aid, as now will be described:

- *Management of incidents*: As discussed in previously, the proposed system is aimed to provide the van driver with an aid in his daily operations. One of the activities that is more prone to error is the loading and unloading of the containers and hence the developed system alerts the driver via a led indicator of its correct execution. However, other incidents that may occur must be taken into account, such as deviations in the estimated route time or the loss of containers in the pharmaceutical stores. It has been established that more than 10% of deviation in the estimated time for the delivery of an order or a non-conformity in the containers to download must generate an automatic incident. When the system detects a lost container, the embedded device sends by Bluetooth to the mobile application how many containers are missing in order to for them to be requested by the store administration. All these incidents are managed by the mobile application, simultaneously alerting both the carrier and control center. This is achieved by using communications established via GPRS/HSPA between the mobile device and the control software solution. The van driver will have the opportunity to see on the Smartphone at any moment which is the state of the route, which issues have been generated, and what are the activities to be undertaken to resolve them. As it can be seen, the mobile application helps the carrier in the development of daily activities, allowing reducing operational errors in the process significantly.
- *Navigation aid*: once in the course of the route and since these are changing according to the pharmacies involved in them, the mobile device offers integrated navigation service for helping the carrier. It shows the route, indicating the order in which the driver must make every stop on the planned route and if it is necessary, it assists delivery man in navigating from one point to another in that route.
- *Support for transport activities*: at each stop, the onboard system reads RFID tags and detects changes in the cargo that are sent via Bluetooth to the mobile application. This data relating to the operation of the carrier in the loading or unloading of containers, provides real-time information about possible deviations (human errors in cargo management) allowing warehouse staff to rectify the errors on delivery in minor time.

### 4.3. Control Software Solution

The control software solution relates to an application for monitoring medicine traceability, schedule optimized routes and locate different vehicles of the vans fleet. This includes the development of a control panel with three main functional features:

- *Medicine traceability*: the system has a robust database where all delivery information is stored, *i.e.*, pharmacy office in which each medicine unit has been distributed, indicating batch number and expiry date. It allows user to search for a container even if it has been downloaded in a pharmacy office or if it is inside a van during a transport service.
- *Fleet management*: the system can locate different distribution vehicles on a map, it can store completed routes and the time spent on each stop. It contributes to the distribution company calculating an approximated time left to deliver a batch on a pharmacy office. It also calculates optimized routes taking into account delivery time, traffic and preemptive supply.
- *Optimized schedule fleet*: taking into account database stored information, using artificial intelligence techniques and the data provided by the ERP, the application generate routes for each vehicle optimizing the time of delivery.

The architecture of the control software solution installed at the servers of the central office is divided into functional layers following a modular structure that facilitates reuse, minimize the coupling and allow future functional enhancements to the platform, Figure 12.

Thus, different functional modules can be distinguished, whose characteristics are described below:

- *Business and service layer*: This module contains all the logic needed to meet the functional requirements previously stated. In addition it manages and gives the necessary permissions for the users of the system. It also manages the data relative to the geolocation of the routes, obtained through external services, in this case using the Google Maps Javascript API.
- *Persistence data layer*: In this layer data that should be stored by the application has been conceptually modeled. Microsoft SQL Server 2008 DBMS has been used in this context. The process of storing the operational data of transportation occurs at the end of the route. When this occurs, the embedded device is connected via WIFI to the server and sends a generated XML file which includes both the actual path followed by the vehicle and the incidences that may have occurred. This file is automatically treated by the system, generating the necessary entries in the DBMS so that the route is recorded at the time of its completion.
- *Communications layer*: Information relevant to the application is in the DB; however, access to such information is done through web services developed with Windows Communication Foundation (WCF) technology. This technical decision allows that both data and application logic can be accessed from other devices, thereby ensuring the scalability and interoperability of the whole system. Security also is increased because the data access does not occur directly but through the services, providing greater control over database queries. The network will be controlled at all times under a firewall that prevents unauthorized access. The set of services developed allows full interoperability between the different components of the system, which is a major benefit in broadening the number of devices compatible with it and enabling their development in the future.
- *Web presentation layer*: the development of the control solution is completed with the control panel web application which offers features beyond the typical application of fleet management. This application has been developed taken into account two fundamental characteristics: (1) maintain a friendly and attractive interface, (2) without prior installation or further configuration. The control panel, based on asp.NET development framework, has been implemented, making extensive use of technologies designed for creating Rich Internet Applications (RIA): JavaScript, CSS3, HTML5, Ajax and jQuery, along with the use of the tools offered by Google for displaying and processing of geographic and positioning information, Figure 12. This feature improves not only the final visual aspect of the application but also the overall usability. The whole site is based on an asynchronous behavior, so interaction eliminates the sense of loading data and responds instantly. All kind of choices as routes, stops or containers represent a dynamic and transparent loading of data and an almost immediately response to their interaction.

## 5. System Validation

**Figure 13.**
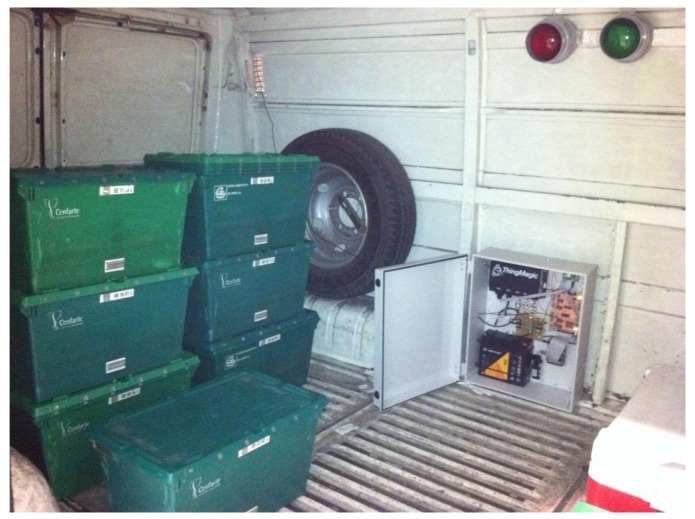
Onboard system deployed on a vehicle. The transport bins of green color with the corresponding RFID tags can be seen on the left hand side of the picture.

The solution has been tested in a pharmaceutical warehouse located in northern Spain. The embedded system has been deployed in a delivery vehicle owned by the company Cenfarte S.A. Figure 13 shows the onboard system installed inside this vehicle for testing purposes. The vehicle has an associated route that takes place up to three times a day. The route includes a maximum of 12 pharmacies and covers a distance of 26.4 km, when all pharmacies are included in the route. The tests were carried out for six days on which the route has been travelled 14 times. The average duration of the route during the tests was 1 h and 37 min.

**Figure 14.**
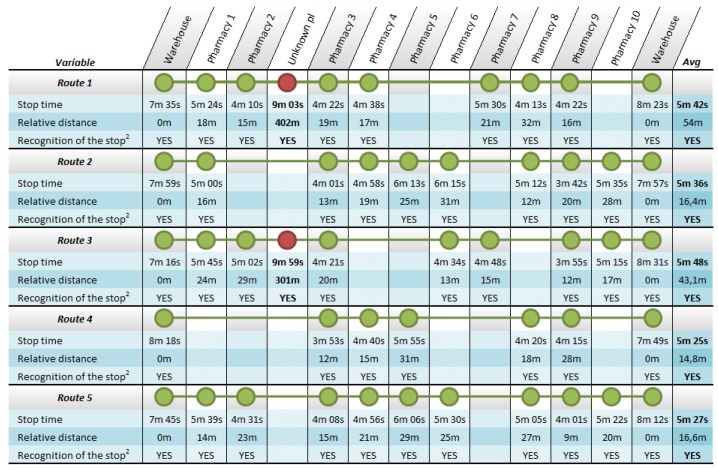
Results of the first phase of system validation: the positioning system. In the table, stop time row shows the duration of the stop from the van parked until the route resumes; relative distance row shows the distance between the position of the van at each stop and the nearest pharmacy on route; and recognition of the stop row indicates whether the stop is identified with the associated pharmacy.

To minimize the impact of the tests in the warehouse, the system evaluation was carried out in three phases. In a first stage the positioning system has been tested. This test was developed during the first two days of validation with five iterations of the route. The system has been continuously storing the location of the vehicle along the route. The information collected in this phase has been used to debug the recognition system of stops and their assignment to pharmacies included in the XML file with the planning of the route. In this stage the definition on how the system has to operate in areas without GPS coverage has also been performed. During one of the developed test routes, an extra stop was added to check the performance of the recognition system. In order to validate the system, an additional stop has been introduced and the corresponding incidence was logged into the XML file. As a result of this test, given the geographical nature of each pharmacy, some of which are located in pedestrian areas where it is impossible to park the Intelligent Van, highlighting the need to modify the structure of the database, including a field indicating the maximum parking distance for each pharmacy. As shown in Figure 14, which reflects the results of each route development in this phase of the system validation, parking distance varies significantly between pharmacies. This inhibits the allocation of each planned stop points in the route, especially when they are close and the detection of non-planned stops during the route performance.

**Figure 15.**
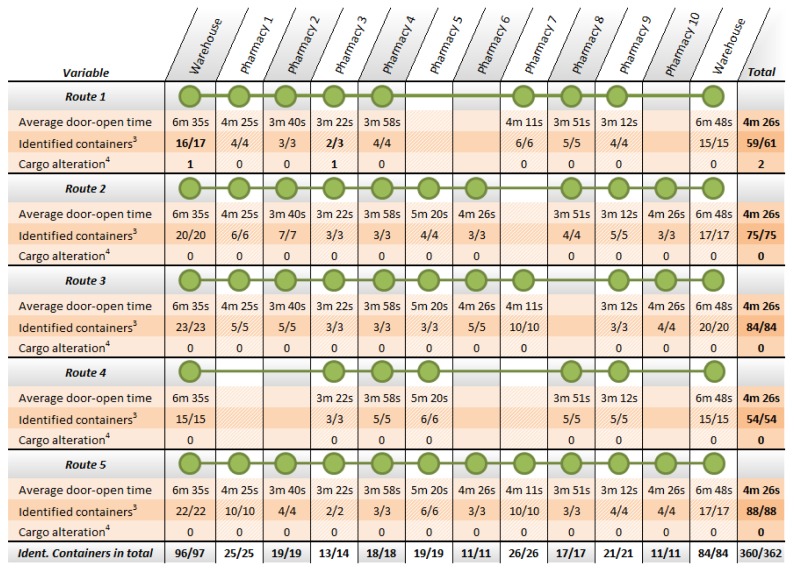
Results of the second phase of system validation: the identification system. In the table, average door-open time row shows the period in which the doors are open at each stop; identified containers row shows the number of containers identified in each stop with respect to the planning; and cargo alteration row indicates mismatches in the load to the schedule set.

In a second stage, tests have focused on the system for identification of the cargo. This test was conducted for six iterations of the route. During this test, the system has stored each cargo alteration detected during periods when the vehicle door remained open. During the development of this test 97 containers have been transported to the pharmacies included in the route. Similarly, 84 empty containers have been loaded and transported back to the warehouse. The analysis of the collected information has reported only one discordance with the actions carried out during the test. This error was due to an incorrectly labeled container affecting delivery at the 3^rd^ pharmacy, as seen in Figure 15. It is worth noting that the system detects this change in the expected load and immediately informs the driver by switching the red traffic light, but corresponding incidence is reported at the exit from the warehouse.

**Figure 16.**
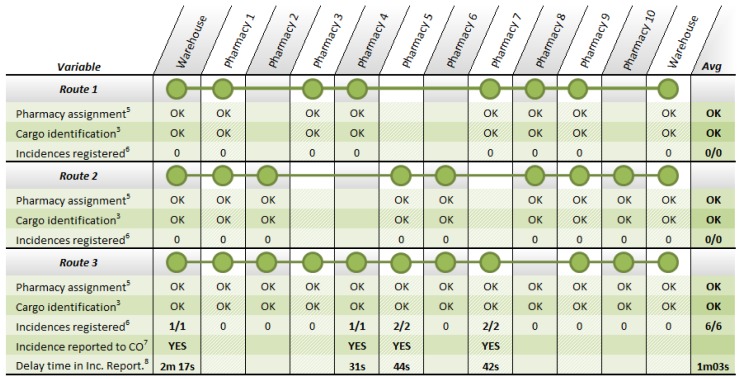
Results of the third phase of the system validation: the complete system. In the table, pharmacy assignment row indicates whether the stop is correctly associated to the corresponding stop; cargo identification row shows if the load carried at the stop is adjusted to the planning; and in incidences registered row, when the system detected an urgent incidence, the correct reception and the period from the reception until the reporting in the control center, are indicated.

Finally, the complete solution was tested during the development of three iterations of the route in the two last days of the system validation (Figure 16). In the first two iterations of the route, the courier was called to pay the maximum attention during the delivery in order to analyze the normal development of a route. In the 17 stops carried out during these first two iterations of the route, the system confirmed the process carried out by the courier, switching on the green light that validates the task. During the execution of the last repetition of the route undertaken to validate the system, the XML file containing the route information was altered manually including three differences with the order given to the courier. Changes included a container that was not ready at the dock of the vehicle (affecting delivery at the 4th pharmacy too) and the exchange between two pharmacies (5th and 7th in the figure) of two containers. The three incidents were detected and reported to the responsible for the warehouse by SMS and email.

## 6. Conclusions

The benefits that item-level traceability for pharmaceutical drugs provides to society in terms of public health and ensuring access to medicines enforces governments to require this feature of the different actors involved in the pharmaceutical supply chain in the short term. The reduction of profit in the pharmaceutical industry motivated by the imposition of certain public policies worsened because of the economic crisis affects not only the laboratories but also distributors of pharmaceutical products that are unable to afford the investment needed for these systems. Most of the initiatives to apply telematic technologies in order to fulfill requirements imposed by governments are being designed without considering the difficulties of deploying such systems in the warehouses currently in operation and in their impact in the activities of the pharmaceutical supply chain.

In this paper we have presented a system based on an intelligent van to improve the distribution of pharmaceutical drugs. The system is able to trace medicines over the delivery routes from warehouses to pharmacies, reporting incidences to carriers in case of anomalies in the distribution plan. This contributes, first to the reduction of the occurrence of errors during distribution and the required time for their recovering and second to locate a set of medicines in case of a mislead.

In order to achieve these tasks, the intelligent van has to identify its environment, including: its location, assets which are contained within the vehicle and the current delivery route. To meet the previous demands, current wireless technologies have been employed: RFID to provide with the cargo identification; GPRS/HSPA, WIFI and Bluetooth to achieve communication; and GPS for the geo-positioning provision. Moreover, interaction with the user has been provided through the integration of a Smartphone in the system.

In order to implement an optimized physical layer interface, detailed analysis of the complex electromagnetic environment given by the indoor cargo area of the vehicle loaded with transporting bins has been performed. Deterministic 3D ray launching simulations have been performed, by means of in-house developed code, which are key in order to estimate wireless link balance and hence performance of the on-board RFID system. The topology of the reader antenna configuration, the distribution of the bins and the properties of the tags can be optimized in order to increase readability and therefore reduce errors. This information has also been used in order to validate and propose a novel embedded antenna configuration, increasing the overall performance of the system.

One of the main contributions of this work is the use of telematic technologies for providing intelligence to a van; this is to improve the distribution of pharmaceutical drugs without altering the way carriers perform their normal tasks. Carriers using the intelligent van will not have to worry about registering (loaded or unloaded) pharmaceutical drug containers; they require no continuous supervision because the system validates every task they make, notifying them only in case of a deviation according to the planned route. It is a non-intrusive solution representing a successful case in using smart environments to resolve a real industrial need.

This has been possible due to first, the design of the technological solution and second, the characteristics of the scenario in which the systems is deployed. The “Good Distribution Practices for Pharmaceutical Products” drafted by the World Health Organization states that all pharmaceutical products should be stored and distributed in containers with no adverse effects on the quality of products, and offering adequate protection from external effects. Thanks to the standardization and reuse of these containers, the transportation of drugs is an ideal scenario for the implementation of RFID tags. The actual high cost of UHF tags is well amortized by a provided application capable of geo-locating the precise position of lost containers, allowing the costs of purchased tags to be affordable in a very short time investment, avoiding one of the biggest drawbacks of this technology (the cost). To summarize, by considering a holistic approach, from radiofrequency physical layer of the RFID system up to the System Architecture definition, a flexible, scalable and competitive solution for pharmaceutical drug delivery has been developed.

## Figures and Tables

**Figure 12.**
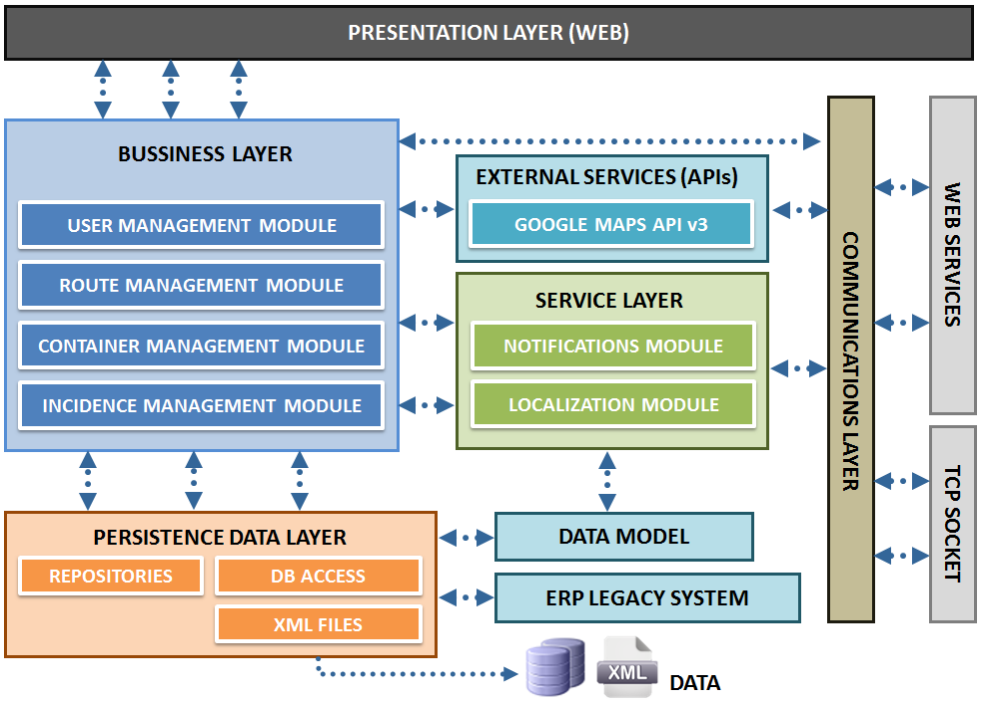
Architecture of the control software solution.

**Figure 12.**
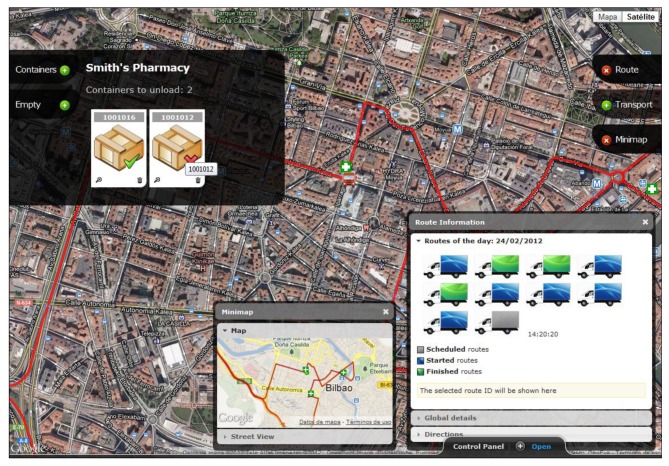
Web interface of the control software solution.
